# Community Pharmacists’ Knowledge in Managing Minor Ailments: A Focus on Childhood Gastroenteritis in Saudi Arabia Using a Simulated Patient Approach

**DOI:** 10.3390/healthcare12232367

**Published:** 2024-11-26

**Authors:** Haifa A. Fadil, Hani M. J. Khojah, Fahad Alzahrani, Ehsan A. Habeeb, Riham Mohamed Elshafie

**Affiliations:** Department of Pharmacy Practice, College of Pharmacy, Taibah University, Madinah 41477, Saudi Arabia; hkhojah@taibahu.edu.sa (H.M.J.K.); fzahrani@taibahu.edu.sa (F.A.); ehabeeb@taibahu.edu.sa (E.A.H.); rshafie@taibahu.edu.sa (R.M.E.)

**Keywords:** community pharmacists, prescribing, minor ailments, acute diarrhea, Saudi Arabia

## Abstract

**Background:** Community pharmacists are frequently approached by patients seeking health advice for minor ailments, particularly for common childhood diseases like diarrhea. Globally, approximately two million children under five years of age die each year due to diarrhea, which remains a significant health concern, especially in developing countries. **Objectives:** The purpose of this study was to assess the skills of community pharmacists in addressing and prescribing for simple viral diarrhea in children. **Methods:** A hundred community pharmacies were visited by simulated clients following a standard scenario of inquiring about simple childhood diarrhea. Subsequently, they filled out a standardized form after each visit to assess the skills of the community pharmacists. **Results:** It was found that 98% of the pharmacists were males. Approximately 80% of them inquired about the child’s age, while only 29% asked about the presence of fever. Around 2–6% of them only asked about the stool nature, child’s feeding behavior, and family symptoms. Around 10% of them suggested a potential bacterial origin, and 24% recommended the use of antibiotics. Only 43% of the community pharmacists suggested the use of oral rehydration solution, while 15–56% recommended using antidiarrheals, anti-emetics, and spasmolytics. The mean knowledge score of the pharmacists was 9.06 out of 17. **Conclusions:** The results indicated a relatively low level of knowledge about managing simple viral childhood diarrhea, which may reflect a similar level of knowledge about dealing with minor ailments in general.

## 1. Introduction

Before non-medical prescribing emerged, only physicians and dentists could prescribe medications. However, the increased prevalence of chronic and mild illnesses has placed a heavy burden on medical staff at primary healthcare institutions [1,2,3]. This has necessitated the adoption of health policies to permit other medical professionals, including pharmacists and nurses, to prescribe medications from a dedicated formulary after receiving formal training and legal authorization [4,5,6]. Over the past few decades, the pharmacist’s role has evolved from medication-centered services, such as compounding and supplying medicines, to the provision of patient-centered care. This has created a new set of responsibilities including disease screening, medication management, patient education, transition of care, medication prescription, and health promotion [7,8,9,10]. This recognition of pharmacists’ role has provided them with the authority to have prescription privileges in the community and outpatient settings [10,11].

Pharmacist prescribing involves three models differing in independence levels: the independent, the collaborative, and the supplementary [5]. In the independent model, pharmacists handle diagnosis, patient assessment, and prescription entirely. The latter two models share a common component in which pharmacists prescribe medications under an independent prescriber who diagnoses and assesses the patient’s condition (a physician or a dentist). In the supplementary model, pharmacists can modify treatment based on a specified clinical management plan agreed upon by the independent prescriber and the patient [5]. A systematic review of 65 studies has identified some factors that motivate pharmacists’ prescribing, which include enhancing patient management, boosting professional confidence, cost reduction, and improving the clinical role of pharmacists [12]. Moreover, several other studies have highlighted other benefits such as enhanced patient outcomes, reduced physician workload, increased pharmacist satisfaction, better skill utilization, and improved healthcare accessibility [12]. However, an American study has reported that the lack of support from medical staff is a key barrier to pharmacists’ prescribing. The main facilitators included support from physicians and senior administrators, training, and enthusiasm [13].

Over the last 30 years, pharmacy practice in the Kingdom of Saudi Arabia (KSA) has undergone significant development. Postgraduate residency training programs have become available, and about 28 pharmacy schools now offer the Doctor of Pharmacy (Pharm. D.) and/or the Bachelor’s (BSc. Pharm.) degrees [14]. Clinical pharmacy practice is currently well founded and supported by the American Society of Health-System Pharmacists (ASHP), particularly in tertiary care hospitals [14]. At the institutional level in KSA, a collaborative agreement between physicians and clinical pharmacists, who have completed postgraduate training, has enabled clinical pharmacists to prescribe certain medications and request laboratory tests in cardiology, anticoagulation, and ambulatory care clinics. However, the role of community pharmacists remains limited to medication dispensing and patient counseling [12,14]. Nevertheless, community pharmacists are frequently approached by patients seeking health advice for minor, or even major ailments, for the sake of cost and time saving [15]. Diarrhea, as a common childhood disease, is an example of this health-seeking behavior [16,17].

Globally, approximately two million children under five years of age die each year due to diarrhea, which remains a significant health concern, especially in developing countries [16]. Rotaviruses account for about 40% of the acute diarrhea cases in the first five years of life, while adenoviruses and noroviruses contribute to approximately 30%. Bacterial pathogens only account for 20% of the cases (e.g., *Campylobacter jejuni*, *Yersinia*, *Salmonella*, *Shigella*, pathogenic *Escherichia coli*, and *Clostridium difficile*) and less than 5% are due to parasitic infection (e.g., *Giardia lamblia*, *Cryptosporidia*, and *Entamoeba histolytica*) [17]. Diarrhea in children can lead to growth failure, cognitive decline, physical impairment, and reduced school performance [16,18]. Furthermore, improper feeding practices, such as formula feeding, can increase vulnerability to diarrhea-causing pathogens [18,19].

As dehydration due to diarrhea is the primary cause of mortality and morbidity in children, the oral rehydration solution (ORS) that contains electrolytes and glucose is usually used to treat diarrhea of any etiology in all age groups [20]. According to the World Health Organization (WHO) and the Saudi Ministry of Health, mild and moderate childhood diarrhea can be managed at home with ORS while avoiding milk, fatty foods, and sugars. Breastfeeding should continue for breast-fed infants, while formula-fed infants require lactose-free formulae. For children on a mixed diet, snacks like bananas, potatoes, boiled apples, jelly, or boiled rice can be introduced after 24 h of rehydration with juices and liquids. Zinc supplement, which is recommended by the WHO, can reduce episode duration by 25% by decreasing stool volume. Severe cases with fever necessitate hospital referral for intravenous fluid administration [21,22].

Although childhood gastroenteritis can result in severe complications such as dehydration and potentially death, it is frequently regarded as a minor condition in community pharmacy practice when exhibiting modest symptoms. This classification indicates pharmacists’ capacity to offer preliminary management recommendations for mild cases, including the suggestion of oral rehydration solutions and dietary modifications. Nonetheless, the importance of early intervention is paramount to avert the advancement to more severe consequences.

Even with the widespread availability of oral rehydration solutions (ORSs) and other advancements in the treatment of childhood diarrhea, there are still gaps in the management of this condition, especially in community pharmacy settings. Pharmacists play a vital role in the provision of primary care for minor ailments; however, research indicates that their practices and knowledge frequently deviate from the established guidelines [15,23]. In Saudi Arabia, where the prevalence of diarrhea in children is estimated to be 25%, evaluating community pharmacists’ competency is important for enhancing patient outcomes and aligning practices with worldwide guidelines [24]. This study aims to address this gap by assessing pharmacists’ knowledge and management of childhood diarrhea as a model of minor ailments through a simulated client methodology in Saudi Arabia.

## 2. Methods

This research was designed as a cross-sectional observational study, adopting a simulated patient methodology to evaluate community pharmacists’ knowledge and management practices for childhood gastroenteritis in Saudi Arabia. A cross-sectional observational methodology was selected because it offers insights into the actual practices of community pharmacists at a particular moment in time. When paired with the simulated patient methodology, this strategy minimizes biases like social desirability bias and recall while capturing real-life encounters. It is particularly effective for assessing the behaviors of healthcare providers without disrupting the natural pharmacy workflow [23,25]. The research was carried out in Madinah, an important city located in the western region of Saudi Arabia with an estimated population of around 1.5 million [26]. Madinah, as a major urban center, features a diverse population and numerous community pharmacies, making it an appropriate context for evaluating pharmacists’ knowledge and practices in the management of minor ailments. The study was conducted between September and October 2024. Selected independent and chain community pharmacies were visited by simulated clients who were trained to follow a standard scenario and complete a standard form after each visit.

### 2.1. Selection of Pharmacies

One hundred private community pharmacies were randomly selected in Madinah. If a pharmacy was closed, it was replaced by another randomly selected pharmacy that was not among the primary selection. A sample size of 100 pharmacies was selected based on practical considerations, including resource availability and the necessity for a representative sample within the study area. Comparable research evaluating community pharmacy practices have utilized similar sample sizes, suggesting that such a size is adequate for obtaining meaningful insights. Ibrahim et al. (2018) conducted a simulated patient study involving 100 pharmacies to assess diarrhea treatment and counseling at community pharmacies in Baghdad [23]. Another study by El Hajj et al. (2011) involved 105 pharmacies in Qatar to evaluate counseling practices for minor ailments [25]. Research indicates that a sample of 100 pharmacies is sufficient for obtaining meaningful data on pharmacist practices; however, subsequent studies may benefit from larger samples to improve generalizability. In selecting the 100 pharmacies for this study, a randomized sampling method was used to ensure a representative distribution across the area. Private community pharmacies were selected as they serve as the principal source for over-the-counter (OTC) medications in Saudi Arabia, providing enhanced accessibility for the general population. Since public pharmacies are typically found in hospitals and primarily serve prescription needs, private community pharmacies are a more appropriate context for evaluating over-the-counter management practices. The inclusion criteria required that pharmacies be community-based and operational during the study period. The exclusion criteria involved hospital-based pharmacies, specialty pharmacies, and any pharmacies that were either temporarily closed or did not provide over-the-counter (OTC) services. This method captured a variety of community pharmacy practices while remaining relevant to OTC patient interactions.

### 2.2. The Simulated Clients

Last-year pharmacy students were trained as simulated clients who followed a standard scenario in collecting the data. This method was adopted because it is more efficient than self-administered surveys for the purpose of this study [27]. In order to guarantee consistency, experienced faculty members from the College of Pharmacy instructed the students who were participating as simulated clients on the study protocol and standardized interaction techniques. Participation was voluntary, with the students receiving academic credit as an incentive, acknowledging their contributions to research and practical learning in pharmacy practice.

### 2.3. The Simulated Client Scenario

The simulated clients entered the pharmacies with the pretense of purchasing medications. Each client was accompanied by a colleague whose mission was to assist the client in remembering the pharmacists’ responses. They approached the pharmacist, inquiring about their young brothers who had been experiencing symptoms of vomiting, diarrhea, and stomachache since yesterday.

The following information was provided if the pharmacist asked for more details. The younger child is eighteen months old, while the elder is four years old. Their temperature is 38 °C and they have no other symptoms. They are engaging in physical activities yet refraining from consuming food or liquids due to vomiting and/or diarrhea.

Other details included crying more often, light brown watery stool, the younger child being formula-fed with occasional breastfeeding, and no similar symptoms in the household. The simulated client then proceeded to provide some practices the mother usually uses to reduce these symptoms such as providing the elder sibling with carbonated beverages (7Up^®^ Keurig Dr Pepper PepsiCo, Burlington, VT, USA) and precooked rice. The simulated client then asked the pharmacist if these practices could help in reducing these symptoms.

Other questions asked by the simulated client included the reason for diarrhea and vomiting, the doses and how to use the medications if anything was recommended, the proper use, doses, and the duration of ORS formulations if they were recommended, any suggested antibiotics, any medications to alleviate the symptoms, whether the younger child should continue breastfeeding or formula feeding, and any other recommendations. Finally, the simulated client departed after apologizing to the pharmacist as if they had forgotten their wallets. Each simulated client then filled in the survey form immediately after leaving the pharmacy.

### 2.4. The Survey Form

The form included basic check boxes for all the planned observations, along with designated areas for further information to be recorded. The initial page included the drugstore’s name and location, the simulated client’s name, and the date and time of the pharmacy visit. Upon completing the form, the simulated client was required to assign a uniform code to each page and thereafter eliminate the first page. The initial papers were gathered by the client leader and secured in a sealed envelope to maintain the researchers’ blindness to the material contained within. Ultimately, the leader gathered all the forms and submitted them to the researchers while preserving a genuine interaction environment.

Additionally, the nationality of each pharmacist was determined during the encounter, as the simulated clients participated in concise, lifelike interactions with the pharmacists. This method allowed the simulated clients to discreetly acquire demographic information, including nationality, as part of the observational data gathered during the study. This design facilitated the unobtrusive capture of pertinent data while preserving a genuine interaction environment.

### 2.5. The Pharmacist’s Knowledge Score

The pharmacists’ knowledge of therapy management for pediatric diarrhea was evaluated using a scoring system that was predetermined and in accordance with standard treatment guidelines [16,28,29]. The pharmacists were evaluated on their knowledge through responses categorized as either correct or incorrect, with an additional option for “no response” based on the given case scenario. Each pharmacist’s response to specific questions asked by the simulated clients was scored as either correct (1 point) or incorrect/unsure (0 points). The cumulative knowledge score ranged from 0 to 21, where higher scores reflected comprehensive knowledge of the recommended practices.

The pharmacists were uninformed about their evaluation, as the simulated client approach was intended to mimic a typical patient interaction. This approach mitigated any bias from behavior modification caused by observation and promoted a more realistic evaluation of the pharmacists’ normal practices and knowledge proficiency.

### 2.6. Ethical Considerations

The study protocol was approved by the Research Ethics Committee of the College of Pharmacy, Taibah University, Saudi Arabia (No.COPTU-REC-110-20240918) on September 2024 with the waiver of informed consent due to inapplicability with the nature of the study, which involved simulated client visits to community pharmacies. The Research Ethics Board approved a consent waiver, as the study aimed to evaluate standard pharmacy processes without disrupting or identifying particular pharmacists. This methodology sought to reduce disturbance and observer bias, ensuring a more realistic evaluation of standard practices. The personal data of the pharmacy staff was not gathered. Pharmacy names were kept confidential unless requested by the authorities.

### 2.7. Data Analysis

Descriptive statistics, such as frequencies and percentages, were employed to summarize the pharmacists’ responses to each observation item in the survey results. In order to evaluate the pharmacists’ competence in treating pediatric gastroenteritis, the mean and standard deviation of the total knowledge score were also determined.

## 3. Results

The results of this study provide an overview of the sex distribution and the nationality counts within a sample of 100 pharmacists. Among the participants, the majority were males, comprising 98 pharmacists (Table 1). In terms of nationality, the highest representation was from Egypt, with 87 pharmacists. Other nationalities included six Sudanese, three Saudi Arabians, two Jordanians, one Yemeni, and one Syrian.

The majority of the pharmacists (81%) asked about the patient’s age (Table 2). A smaller number of them inquired about stool color, the presence of watery stool, and the nature of an infant’s milk feeding. Family symptoms were mentioned the least.

Regarding the diagnosis and treatment recommendations for the pretended cases, it was found that the pharmacists’ assessments of the conditions varied, with bacterial conditions being slightly more frequently identified (10 pharmacists) than viral ones (6 pharmacists). Additionally, 84% of the pharmacists did not assess whether the condition was caused by an infection (Table 3). Notably, oral rehydration solutions (ORSs) were recommended by only 43% of the respondents, which is consistent with standard guidelines. Conversely, 24% of the respondents prescribed antibiotics, which is in contradiction to the most effective treatment for viral diarrhea. Unconventional cures such as carbonated beverages (25%) and precooked rice (43%) were often recommended, illustrating the impact of cultural concepts on recommendations. Surprisingly, only a small fraction of the pharmacists (5%) advised taking ORS ad libitum, and even fewer (3%) recommended mixing ORS with juice and adding extra water to the powdered ORS formula. The pharmacists exhibited a higher propensity to suggest antidiarrheals (56 pharmacists) and anti-emetics (47 pharmacists) as compared with antibiotics (24 pharmacists) and antispasmodics (15 pharmacists). Additionally, a moderate number of the pharmacists advocated continuing breastfeeding (16 pharmacists) and advised consulting a pediatrician (42 pharmacists).

The scoring system used to assess the pharmacists’ knowledge in managing childhood viral diarrhea was structured around 21 key items, each aligned with the recommended practices for diarrhea management, as shown in Table 4. The pharmacists were awarded one point for each correct response, reflecting adherence to standard treatment guidelines. Zero points were awarded for answers that were unclear or inaccurate, as well as for no answers at all. This resulted in a cumulative knowledge score ranging from 0 to 21, with higher scores indicating greater adherence to recommended practices and a more comprehensive understanding of effective management for childhood diarrhea. The mean of the pharmacists’ knowledge score in this study was 9.06 ± 2.35. The minimum observed value was 3, while the maximum value reached 17. The average knowledge score of the participants in this study was 9.06 out of a maximum of 17, which led to the conclusion that pharmacists show a relatively low level of knowledge.

## 4. Discussion

In this study, a simulated patient scenario was used as a method to assess the skills of community pharmacists in Saudi Arabia in addressing and prescribing for simple viral diarrhea. This methodology is a robust approach for objectively assessing real-world practices in a controlled manner. The study showed that male pharmacists were dominant in community pharmacies, representing 98% of the sample. This could be explained by the female preference to pursue different career paths due to the work patterns at the community pharmacy [30]. Furthermore, the study outlined a diverse mix of nationalities at community pharmacies, with the largest segment originating from Egypt, underscoring the multicultural nature of the population under investigation.

The fact that most pharmacists (81%) inquired about the patient’s age aligns with the notion that age is a significant factor in disease occurrence, susceptibility, and treatment response [31]. However, the lower frequency of inquiries about symptoms such as the patient’s temperature, flu symptoms, stool color, watery stool, and the nature of the infant’s milk feeding may reflect inefficient diagnostic skills. Notably, about 30% of the pharmacists asked about the patient’s temperature and flu symptoms, indicating a significant concern for infectious diseases. However, still, 24% of them recommended the use of antibiotics even though the presented symptoms suggest a viral origin for the infection.

The study also found that a relatively small number of pharmacists asked about gastrointestinal symptoms (e.g., stool color and watery stool) and infant feeding practices. This finding suggests a surprisingly less comprehensive approach to patient assessment, given that gastrointestinal symptoms are common patient complaints in community pharmacies [32].

Similarly, inquiring about infant feeding practices could provide important insights into the possible causes of minor ailments in infants, as feeding practices significantly influence infant health [33,34,35]. On the other hand, the lowest number of inquiries about family symptoms might indicate a missed opportunity for family-focused care [36].

The pharmacist must distinguish between the types and causes of diarrhea in order to effectively deal with the patients. This can be achieved by taking a complete patient history [37]. It is crucial to assess the severity, duration, and frequency, as well as any other symptoms such as fever, dehydration, vomiting, urine output, and thirst. Further investigation may also include the type of stool (e.g., bloody or watery) and whether the patient has any other diseases or has taken any medications recently [23].

The study’s findings also bring to light intriguing aspects of pharmacists’ approaches to diagnosing and treating diarrhea. The slight preference for identifying bacterial conditions over viral ones might reflect the pharmacists’ inclination towards conditions that can be treated directly with pharmaceutical interventions, such as antibiotics. However, this approach might also raise concerns about the potential over-prescription of antibiotics, which could contribute to antibiotic resistance, a primary global health concern [38,39].

Interestingly, the study showed that some pharmacists recommended unconventional treatments such as 7Up^®^ and precooked rice, which may reflect the impact of cultural health beliefs on community pharmacy practice in Saudi Arabia. However, although precooked rice and fluid replacement are recommended to control childhood diarrhea, using soda, energy drinks, and other beverages can worsen it by inducing osmotic diarrhea [40].

Although 43% of the pharmacists recommended the use of ORS, which is in line with the WHO guidelines for managing dehydration resulting from diarrhea [41,42], only a small number of the pharmacists advised using it ad libitum. In addition, some pharmacists (3%) recommended mixing the ORS with juice or extra water, which suggests a potential gap in understanding or communicating the best practices for ORS use.

Antidiarrheals, anti-emetics, and antispasmodics are common over-the-counter medications for minor gastrointestinal ailments [43,44]. However, their use in simple viral childhood diarrhea is not recommended as they may interfere with the natural defense mechanisms. Unfortunately, they were frequently recommended by the community pharmacists (56%, 47%, and 15%, respectively). Likewise, antibiotics are not recommended for this type of diarrhea. However, 24% of the pharmacists recommended using them although they cannot be sold without a formal prescription [39]. On the other hand, breastfeeding is highly recommended, and it protects against morbidity and mortality due to childhood diarrhea [45]. Nonetheless, only 16% of the pharmacists in this study recommended continuing it.

Finally, the mean pharmacist knowledge score of 9.06 out of 17 indicates a relatively low level of knowledge about the management of simple viral diarrhea in children among community pharmacists in Saudi Arabia. Similar research evaluating the knowledge levels of healthcare practitioners have found that a score below 50% of the total possible points is frequently suggestive of limited knowledge [23,25]. Although there is no universally standardized criterion, this threshold offers a practical method for classifying knowledge as either low or high, enabling meaningful interpretation and comparison with other studies. This finding may ring a bell about the low level of understanding of therapy management for minor ailments, especially for pediatric conditions such as diarrhea. Moreover, the findings emphasize the need for focused interventions by highlighting important gaps in community pharmacists’ practices and knowledge regarding the management of childhood diarrhea. Tailored training programs that emphasize evidence-based guidelines, including the appropriate use of ORS and the avoidance of unnecessary antibiotic prescriptions, should be devised and implemented. In addition, pharmacists must receive deeper education and pass more focused and clinically oriented licensing exams before being engaged in patient assessment and minor ailment management. These initiatives would likely ensure better patient outcomes and enhance pharmacists’ competencies.

### 4.1. Limitations of the Study

Although this approach of simulation may be very effective, it is difficult to achieve consistent standardization across all the simulated scenarios. Furthermore, the simulated scenarios may not fully replicate the complexity of real-life clinical situations, where diverse patient presentations and unpredictable interactions may influence pharmacists’ responses. Moreover, this study was conducted in one city in Saudi Arabia, where certain practices exist. This can overestimate the external validity of the study. Furthermore, the sample of this study was primarily composed of male pharmacists from a single nationality, which is reflective of the current demographic composition of community pharmacists in the region. Although this offers valuable insights into the practices of this group, it may restrict the generalizability of the results. Future studies could consider including a larger sample, and more diverse samples across genders and nationalities to enhance the applicability of results to a broader population of pharmacists.

In addition, this study may be influenced by potential recollection bias, as pharmacists’ responses may differ according to their prior experiences. To minimize these biases, we employed strategies including random pharmacy selection and the application of standardized scripts for simulated clients. Finally, certain community pharmacists may have modified their conduct if they perceived that they were being observed. To mitigate potential bias, particular steps were implemented to ensure that the simulated clients resembled typical customers, hence reducing the probability of changing behavior from observed pharmacists. Nevertheless, as the potential for observer bias cannot be completely eliminated, future studies should consider utilizing more discreet observation methods, such as covert procedures, to mitigate this prejudice.

### 4.2. Implication of the Study

Future research should concentrate on the assessment of the efficacy of pharmacists’ targeted training interventions, particularly in the management of a broader spectrum of minor ailments beyond childhood diarrhea. Studies could investigate innovative methods, including simulation-based training, seminars, and e-learning modules, that are intended to improve the clinical competencies and adherence of pharmacists to evidence-based guidelines. Furthermore, research should examine the factors that influence the knowledge and practice of pharmacists, such as their educational background, years of professional experience, and participation in continuing professional development programs. An understanding of these factors can assist in the customization of interventions to address specific knowledge gaps and practice deficiencies. These studies would not only inform the design of targeted educational programs but also influence policy adjustments to integrate these interventions into pharmacy curricula and licensure requirements. Ultimately, these efforts have the potential to enhance the role of pharmacists in primary healthcare, thereby reducing the burden on other healthcare providers and enhancing patient outcomes.

## 5. Conclusions

This study provides a comprehensive snapshot of community pharmacists’ current practices and knowledge in managing minor ailments in Saudi Arabia, particularly childhood gastroenteritis. The findings underscore pharmacists’ crucial role in managing minor ailments and highlight potential areas for improvement. Targeted educational interventions, such as the development and implementation of structured training programs that follow evidence-based guidelines, could significantly improve the competencies of pharmacists, and optimize patient care. These interventions could include the appropriate use of oral rehydration solutions and the avoidance of unwarranted antibiotic prescriptions. In addition, regulatory bodies may integrate mandatory clinical assessments and continuous professional development that concentrate on the management of minor ailments into licensure requirements. To generalize the findings, future research should replicate this study in diverse geographic and demographic contexts, utilizing larger sample sizes. Furthermore, future research could further explore the factors influencing pharmacists’ knowledge scores, such as their educational background, years of experience, and exposure to professional development opportunities. Such research could inform the development of targeted training interventions aimed at improving pharmacists’ knowledge and skills in managing minor ailments effectively. Addressing these gaps through education and policy adjustments may strengthen pharmacists’ contributions to primary healthcare and improve patient outcomes.

## Figures and Tables

**Table 1:** Characteristics of the pharmacist sample (N = 100).

Item	n and %
Sex	
Male	98
Female	2
Nationality	
Egypt	87
Sudan	6
Saudi	3
Jordan	2
Yemen	1
Syria	1

**Table 2:** Pharmacist’s inquiry about the patients’ descriptive symptoms (N = 100).

Item	n and %
Patient’s age	81
Patient’s temperature	29
Flu symptoms	10
Stool color	2
Watery stool	6
Nature of infant’s milk feeding	4
Family symptoms	2

**Table 3:** Pharmacists’ assessments and treatment recommendations for diarrhea (N = 100).

Item	n and %
Assessment of Cause	
Viral	6
Bacterial	10
Unspecified/No Answer	84
Treatment Recommendations	
Suggested using 7Up^®^ as a remedy	25
Recommended eating precooked rice	43
Recommended ORS usage	43
Advised ORS ad libitum	5
Suggested mixing ORS with juice	3
Recommended adding extra water to ORS	3
Prescribed antibiotics	24
Recommended antidiarrheal	56
Recommended anti-emetic	47
Recommended antispasmodic	15
Recommended continuing breastfeeding	16
Advised consulting a pediatrician	42

ORS, oral rehydration solution.

**Table 4:** Knowledge assessment items and pharmacists’ responses for managing childhood viral diarrhea (N = 100).

No.	Item	n and %
1	Inquiry about the patient’s age	81
2	Inquiry about the patient’s temperature	29
3	Inquiry about flu symptoms	10
4	Inquiry about stool color	2
5	Inquiry about watery stool	6
6	Inquiry about nature of infant feeding	4
7	Inquiry about family symptoms	2
8	Stating that the cause is a virus	6
9	Denying that the cause is bacteria	90
10	Denying drinking 7Up^®^ as a remedy	75
11	Recommending precooked rice as a remedy	43
12	Recommending ORS as a remedy	43
13	Recommending ORS ad libitum	5
14	Denying mixing ORS with juice	97
15	Denying adding extra to powdered ORS	97
16	Denying using an antibiotic	76
17	Denying using an antidiarrheal	44
18	Denying using an anti-emetic	53
19	Denying using an antispasmodic	85
20	Recommending continuation of breastfeeding	16
21	Recommending seeing a pediatrician	42

ORS, oral rehydration solution. zero was considered for incorrect answers, uncertainty, or no response.

## Data Availability

Dataset available upon request from the authors.
